# Nemertodermatida—Endosymbionts of Deep-Sea Acorn Worms (Hemichordata, Torquaratoridae)

**DOI:** 10.1134/S001249662360015X

**Published:** 2024-03-12

**Authors:** O. V. Ezhova, A. I. Lukinykh, V. V. Malakhov

**Affiliations:** Moscow State University, Moscow, Russia

**Keywords:** Xenacoelomorpha, *Quatuoralisia malakhovi*, Enteropneusta, histological structure, Bering Sea

## Abstract

Worm-like endosymbionts were found in the hepatic region of the digestive tract of the deep-sea acorn worm *Quatuoralisia malakhovi* Ezhova et Lukinykh, 2022 (family Torquaratoridae) from the Bering Sea. The symbionts were assigned to the taxon Nemertodermatida on the basis of histological examination. Torquaratoridae are similar in feeding type to holothuroids, which have also been found to have Xenacoelomorpha endosymbionts.

The deep-sea acorn worms Torquaratoridae have recently been discovered as a group of marine invertebrates [1–5]. These animals live at depths of 350–8800 m [3, 4, 6] and do not bury in the seafloor in contrast to shallow-water acorn worms. A high population density of Torquaratoridae has been observed in certain oceanic regions. For example, their population density reaches 12 acorn worms per square meter in the Bering Sea at depths of approximately 2000 m [7]. Given this population density, Torquaratoridae certainly play an important role in the function of bathyal and abyssal communities.

The biology of Torquaratoridae is poorly understood. In particular, little is known about their symbionts. Acoelic flatworms and parasitic copepods have been mentioned as gut inhabitants of *Torquarator bullocki* Holland, Clague, Gordon, Gebruk, Pawson and Vecchione, 2005, in the first publication about Torquaratoridae, but without any description or illustration [1]. A white process on the outer side of the genital wings has been observed in an underwater image of *Allapasus isidis* Priede, Osborn, Gebruk, Jones, Shale, Rogacheva and Holland, 2012, and is possibly a leech attached to the worm [4]. Trematode metacercariae have been found in all divisions of the coelom in *Quatuoralisia malakhovi* Ezhova et Lukinykh, 2022 [8].

Here we describe the Nemertodermatida sym-bionts found in the deep-see acorn worms Torquaratoridae.

Material for the study was collected on June 18, 2018, during the 82nd expedition onboard the *Akademik M.A. Lavrent’ev* research ship. Trawling was performed at station LV 82-9 (55.3451–55.3466° N, 167.2750–167.2752° E) in the Komandorsky Basin (the Volcanologists Massif) of the Bering Sea at depths of 1957–1933 m. *Quatuoralisia malakhovi* individuals were fixed with 8% formalin in seawater for histological examination. Specimens were washed to remove the fixative and dehydrated with increasing ethanol concentrations by standard methods. Fragments prepared for histological examination were embedded in Paraplast, and the resulting blocks were used to obtain series of transverse and sagittal 10-µm histological sections with a Leica RM 2125 microtome. The sections were stained with Carracci hematoxylin and eosin (an ethanol solution). Images of the sections were obtained using a Micmed-6 microscope (LOMO, St. Petersburg, Russia, 2018) with a MS-12 digital camera. Five worms were examined, and symbionts were found in two of them.

The infected *Q. malakhovi* specimens were females and carried a single symbiont each. The symbionts were found in the gut lumen in the anterior part of the *Q. malakhovi* hepatic region. The symbionts appeared elongate leaf-shaped in sections (Figs. 1a, 1b). One symbiont was approximately 900 µm long and 400 µm wide in the widest part of its body; the dimensions of the other symbiont were 1 mm in length and approximately 870 µm in width. The symbionts were in contact with folds of the *Q. malakhovi* gut epithelium.

**Fig. 1.**
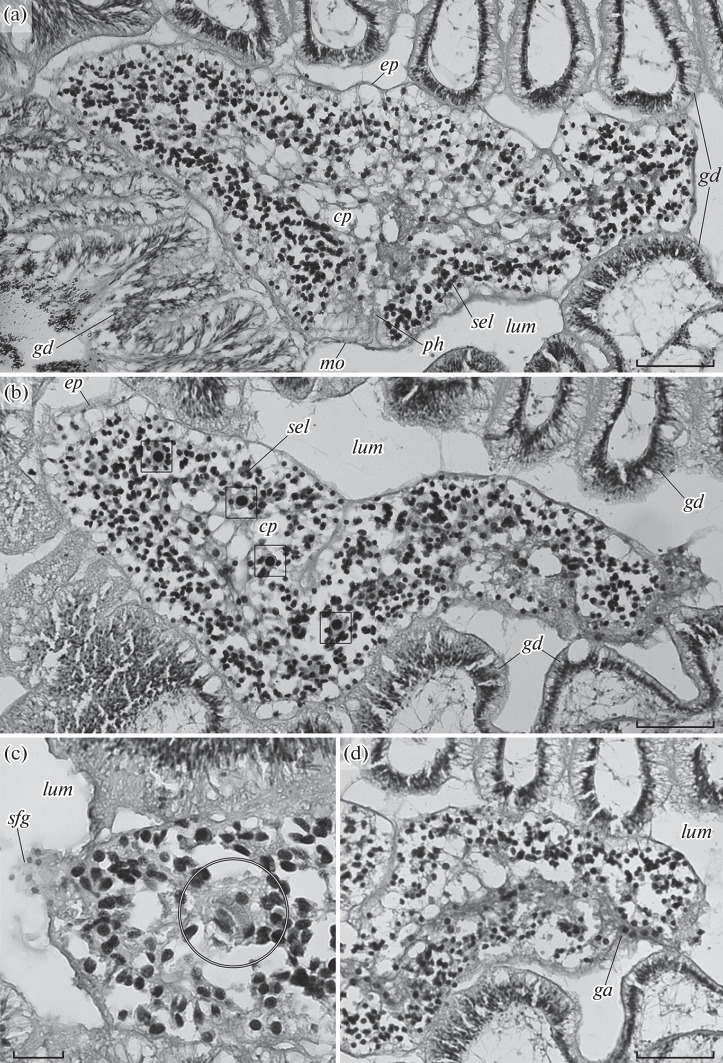
Histological sections of the symbionts within the hepatic region of *Quatuoralisia malakhovi*: (a) a longitudinal section with the mouth (*mo*) and the pharynx (*ph*), (b) a longitudinal section with gonia (square frames), (c) part of a transverse section of the anterior end with a presumable statocyst (circular frame), and (d) part of a longitudinal section with the genital atrium (*ga*). Bar, (a, b, d) 100 or (c) 20 μm. Designations: *cp*, central parenchyma of the symbiont; *ep*, epidermal epithelium of the symbiont; *ga*, genital atrium; *gd*, gastrodermal epithelium of the host; *lum*, gut lumen of the host; *mo*, mouth; *ph*, pharynx; *sel*, subepidermal layer of the symbiont; *sfg*, secret of the frontal glands.

A vacuolated ciliated epithelium of 20 µm in height coats the symbionts (Figs. 1a, 1b; *ep*). A basal lamina underlying the coating epithelium was not detected. A subepidermal layer occurs under the coating epithelium and consists of small cells (Figs. 1a, 1b; *sel*). The height of the subepidermal layer varies from 50 to  80 µm. The central part of the body is occupied by  large vacuolated cells of the central parenchyma (Figs. 1a, 1b; *cp*). A mouth opening is 100 µm wide, occurs in the middle of the body (Fig. 1a, *mo*), and leads to a cylindrical pharynx, which is 130 µm deep (Fig. 1a, *ph*). A statocyst is found at the putative anterior end of the symbiont (Fig. 1c, circle framed). The statocyst is an oval body of 15 µm in diameter and hangs on radial ligaments. Statoliths were not preserved. However, two flattened nuclei are found in the center of the statocyst, presumably corresponding to the nuclei of two lithocytes. Secretion of frontal glands is detectable at the anterior end (Fig. 1c, *sfg*). A genital atrium opens subterminally at the opposite, posterior end of the body (Fig. 1d, *ga*). A dark bundle extends from its opening into the inside of the body and is adjacent to the central parenchyma. Separate large cells with a large nucleus and a dark cytoplasm are found at the boundary of the subepidermal layer and the central parenchyma (Fig. 1b, square framed). The cells can be assumed to be gonia, which are precursors to gametes. Structures that could be assigned to the excretory system were not found in the histological sections of the symbionts. Likewise, nerve and muscle fibers were not distinguished in the sections stained with hematoxylin and eosin.

The main organization features (lack of a basal lamina of the coating epithelium, the positions of the mouth and genital atrium, lack of an excretory system, lack of structured gonads, the oogonia occurring at the boundary the subepidermal layer and the central parenchyma, and a statocyst with two lithocytes) allow us to assign the symbionts to the group Nemertodermatida, which belongs to Xenacoelomorpha together with Acoela and Xenoturbellida. Acoela and Nemertodermatida are considered to be sister groups that form the clade Acoelomorpha [9–12].

Various holothurians mostly predominate in the macrozoobenthos community of the slopes of the Volcanologists Massif at depths of 1370–4278 m [7]. However, the torquaratorid *Q. malakhovi* displaces holothurians from their dominant position at depths of 1830−2290 m [7, 13]. The ecological niches of Torquaratoridae and Holothuroidea overlap because both are detritus feeders [7, 14–16]. It is of interest that shallow-water holothurians are also known to have symbionts that belong to Acoelomorpha and inhabit their digestive tract [17, 18]. For example, *Aechmalotus pyrula* Beklemischev, 1915, which represents Acoela, lives in the digestive tract and respiratory trees of the holothurian *Eupyrgus scaber* Lütken, 1857, from the Barents Sea. The taxon Acoela also includes the members of the genus *Aphanostoma* Ørsted, 1845, that have been found in the digestive tract of the holothurians *Myriotrochus rinkii* Steenstrup, 1851 and *Chiridota laevis* (O. Fabricius, 1780) from the Barents Sea [18]. *Meara stichopi* Westblad, 1949, which belongs to Nemertodermatida, has been found in the digestive tract and coelomic cavities of the holothurians *Mesothuria intestinalis* (Ascanius, 1805) and *Parastichopus tremulus* (Gunnerus, 1767) from the North Sea [17, 19]. Thus, the presence of endosymbionts belonging to related groups reflects the similarity in ecological niches between Holothuroidea and Torquaratoridae.
